# Identifying and modeling built environment factors influencing cultural perception in metro stations: Evidence from central Shanghai

**DOI:** 10.1371/journal.pone.0334642

**Published:** 2025-11-06

**Authors:** Haoxuan Feng, Xuan Xiao, Yue Cheng, Rongbing Mu, Li Xiong

**Affiliations:** 1 School of Design and Art, Jingdezhen Ceramic University, Jingdezhen, Jiangxi, China; 2 School of Architecture and Urban Planning, Tongji University, Shanghai, China; Chang'an University, CHINA

## Abstract

As urban rail transit expands, systematic evidence remains limited on how the built environment influences cultural perception among passengers. This study identifies the main determinants of cultural perception, tests whether perception of nearby public cultural facilities mediates these effects, and examines heterogeneity by station type. Using metro stations in central Shanghai as a case, we compute the Shannon diversity index of nearby public cultural facilities within the 500 m station area and apply Anselin Local Moran’s I to classify 90 stations into four types: High-High cluster, High-Low outlier, Low-High outlier, and Low-Low cluster. Questionnaire data from 12 representative stations (n = 414) are analyzed with structural equation modeling, and differences across station types are assessed with a one-way analysis of variance. Results indicate that interior spatial design satisfaction has the strongest positive association with cultural perception, followed by entrance and exit design satisfaction. Perception of nearby public cultural facilities is positively associated with cultural perception and partially mediates the association between interior spatial design satisfaction and cultural perception. Station types differ significantly in interior spatial design satisfaction, entrance and exit design satisfaction, perception of nearby public cultural facilities, and cultural perception, with High-High cluster highest, Low-Low cluster lowest, and High-Low outlier and Low-High outlier in between. This study incorporates the subjective perception of nearby public cultural facilities into the framework for cultural perception in metro stations, clarifies direct and mediated pathways, and provides type specific implications for factor prioritization and station stratification in upgrades and retrofits across different network contexts.

## Introduction

As of January 1, 2025, urban rail transit operated in 58 mainland Chinese cities, comprising 362 active lines with a cumulative length of 12,168.77 km. Metro lines accounted for 76.27% of this network and remain the backbone of urban public transport [1]. With rapid network expansion, metro stations are shifting from throughput-oriented transport nodes to multifunctional public spaces that combine mobility, social interaction, and cultural communication [2,3]. However, across station types, which station-area built-environment elements influence passengers’ cultural perception remains unclear, hindering targeted cultural-expression design and evaluation.

In recent years, cultural perception in metro environments is grounded in sensory input and meaning interpretation. Corresponding studies, on the one hand, assess the effects of thermal comfort, accessibility, and wayfinding systems on sensory experience [4–6], and, on the other hand, examine how art installations, visual symbols, and thematic imagery shape cultural experience [7–9]. However, much of this literature emphasizes visible aesthetics. Mechanism oriented models that integrate station interior and access with the station area cultural context remain limited, and heterogeneity by station type has received comparatively little attention.

Concurrently, public cultural facilities (e.g., museums, libraries, and cultural centers) function as key nodes in the urban cultural network: they are common trip destinations and can shape the perceived cultural atmosphere of stations. Within the perspectives of transit oriented development (TOD) and the node-place model [10,11], spatial adjacency between stations and such facilities suggests potential for integrated cultural services. Yet most studies remain at the community or neighborhood scale; the mechanisms through which facilities within a station’s service area influence passengers’ cultural perception are still insufficiently examined [12,13], with limited attention to structural attributes such as public cultural facility type diversity and the evenness of spatial distribution.

Accordingly, this study addresses three questions: (1) In a structural model, do the three station core built environment dimensions (interior spatial design, entrance and exit design, and service facilities) and perceptions of nearby public cultural facilities each make an independent contribution to cultural perception? (2) Does nearby public cultural facilities perception mediate the associations between the three station core dimensions and cultural perception? (3) Do different station types differ in cultural perception. Neglecting the holistic cultural context that links the station and its surroundings risks fragmented cultural expression and piecemeal strategies that limit the cultural value of transit spaces.

We investigate central Shanghai by combining point of interest (POI) records with a passenger survey to conduct an empirical analysis along two dimensions: spatial structure and perceived experience. Facility diversity is measured and spatial clustering is applied to identify representative stations. A structural equation model is then estimated to test pathway relationships and mediation among the three station core dimensions, perception of nearby public cultural facilities, and cultural perception. Station-type differences are examined through group comparisons within the same analytical framework.

This study makes two contributions. First, it positions nearby public cultural facilities as a station area factor within a mechanism for metro cultural perception, thereby extending the analytical scope of cultural perception research. Second, it provides empirical evidence that satisfaction with interior spatial design, satisfaction with entrance and exit design, and perceptions of nearby public cultural facilities make independent contributions to cultural perception, that nearby public cultural facilities perception partially mediates the link from interior spatial design to cultural perception, and that cultural perception differs by station type. These results offer theoretical support and practical implications for cultural expression in metro environmental design.

## Literature review

### Concepts and theories of cultural perception

Perception originates in psychology and denotes the mental process whereby external stimuli are received by the sense organs, transduced into neural signals, and processed and interpreted by the brain [14]. Cultural perception extends perception into cultural contexts: external objects or environments act as cultural carriers or ambient settings that, via sensory input, elicit psychological responses and shape individuals’ understanding and impressions of a specific area [15].

In environmental and sustainability sciences, cultural perception is often articulated within the cultural ecosystem services paradigm and understood as the cognitive interpretation and appraisal of nonmaterial or meaning based values (e.g., aesthetic, symbolic, sense of place, and memory) arising from human environment interactions [16]; In cultural heritage research, it is used as an evaluation criterion for the conservation of intangible values (including symbolic meaning, historical value, and social memory) based on public perception of and identification with cultural significance [17]. In urban planning and the built environment literature, cultural perception is operationalized as the interpretation of cultural cues embedded in urban form and public space, informing place meanings and aesthetic judgments [18–20].

Cultural perception differs from spatial perception: the latter concerns the representation of physical attributes such as scale, structure, and materiality [21,22], whereas the former concerns the meanings and affective ambience attached to those attributes within cultural frames. With respect to cultural cognition, cultural perception refers to the sensory uptake and on site interpretation of cultural cues, while cultural cognition denotes knowledge structures (schemas, beliefs, and values) that organize interpretation, memory, and reasoning across situations; cultural cognition can guide perception through expectations and categorization but is not the perceptual experience itself [23–25]. With respect to cultural image, cultural perception is a momentary, observer specific appraisal during encounters with the built environment, whereas cultural image denotes a shared and relatively enduring mental representation of a place’s cultural characteristics formed through accumulated perceptions and communicative processes, and is therefore more outcome level and collective [18,26]. With respect to place attachment, cultural perception is a context specific cognitive and affective appraisal of cultural information, whereas place attachment is a more stable emotional bond with a place that develops over time; relatedly, place identity represents the cognitive identity dimension linked to but distinct from attachment [27,28].

### Built environment at metro stations

In the urban design and transport planning literature, the built environment of public buildings (including transport facilities) typically encompasses the building and its interfaces, as well as adjacent spaces and facilities within the station area [29,30]. In travel behavior and station area studies, the built environment is further framed as a composite of the node place model and the 3D and 5D dimensions, an external context jointly constituted by network accessibility and land use intensity, diversity, and design quality [11,31]. Accordingly, the built environment of metro stations can be understood as comprising the station core and the station area.

For the station core, prior research shows that station design shapes transfer performance (e.g., transfer time, walking distance, legibility) and thereby the perceived transfer penalty and overall user experience [32,33]; interior color and lighting, wayfinding systems, and surface materials affect visual comfort and can trigger associations [34,35]; and metro stations function as perceptual nodes of the urban image, amplifying or attenuating place specific cultural cues [36]. These findings imply that on site elements influence not only spatial behavior but also the encoding of cultural information through symbolism and ambience.

For the station area, walking to stations is jointly influenced by the perceived built environment, distance, and travel attitudes [37]. Environmental characteristics within the station area are associated with users’ emotions and perceived quality [38,39]. In other words, the station-area context determines when, where, and along which routes passengers encounter exogenous cultural cues, shaping their salience and interpretation.

### Public cultural facilities in the station area

Building on the above station area framing, we focus on nearby public cultural facilities (e.g., libraries, museums, galleries, cultural centers) as a major component of the built environment of station area. The station area is typically delineated by walking distance, with buffer radii of 400 m, 700 m, or 800 m commonly used [40–42], and a 500 m buffer widely adopted in studies of Asian cities [43].

Within station area research, nearby public cultural facilities are commonly discussed in relation to spatial density, facility type diversity, and network accessibility. Facility type diversity is often measured using indices such as the Shannon diversity index and aligns with heterogeneous cultural needs and participation preferences, thereby expanding activity choices [44,45]. Regarding accessibility and participation, proximity and public transport connectivity to cultural venues are associated with higher attendance, whereas distance and travel cost reduce visitation [46,47]; Improving the spatial balance of public cultural facilities can better meet residents’ cultural needs [48]. Over time, the siting and accessibility of public cultural facilities evolve with urban development and planning policies, reshaping spatial access and the conditions under which visitation occurs [49]. In addition, exhibitions and sensory experiences offered by museums and cultural centers can shape visitors’ or residents’ place identity and the urban image, thereby influencing the perception and appraisal of cultural meanings [50,51]. Taken together, these findings suggest a connection between nearby public cultural facilities and cultural perception.

### Theoretical framework and hypotheses

This study situates cultural perception within a built environment framework. Drawing on passengers’ evaluations and perceptions of physical attributes inside and around metro stations, we identify the contributing factors and organize them into a structural pathway model.

**Fig 1:**
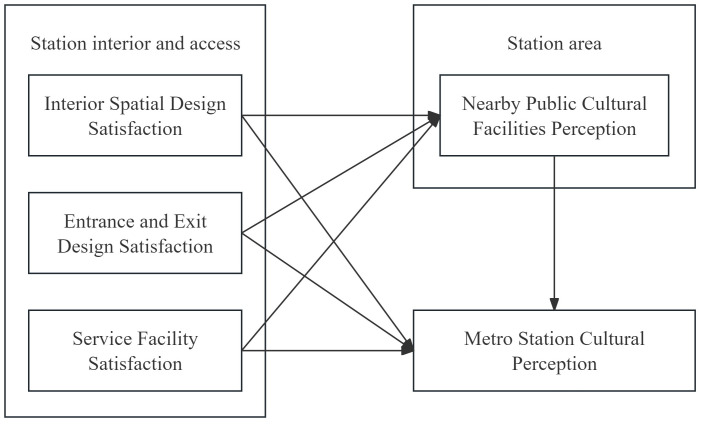
Conceptual pathway model of station cultural perception. Created by the authors.

Building on the preceding literature review, we propose a conceptual model of cultural perception in metro stations (Fig 1). The model comprises five latent constructs: three dimensions of the station interior and access (interior spatial design satisfaction, entrance and exit design satisfaction, service facilities satisfaction); nearby public cultural facilities perception representing the built environment of station area; and overall station cultural perception. The model specifies (1) direct paths from the three dimensions to cultural perception, (2) a direct path from nearby public cultural facilities perception to cultural perception, and (3) indirect paths whereby the three dimensions influence cultural perception through nearby public cultural facilities perception. Consistent with the literature summarized above, we conceptualize perceived nearby public cultural facilities as a proximal, content-specific appraisal that transmits the effects of interior and entrance/exit design to overall cultural perception; accordingly, we treat it as a mediator rather than a moderator in the model. These assumptions lead to the following hypotheses (H1–H7).

H1: Satisfaction with interior spatial design exerts a significant positive effect on cultural perception.

H2: nearby public cultural facilities perception exerts a significant positive effect on cultural perception.

H3: Satisfaction with entrance and exit design exerts a significant positive effect on cultural perception.

H4: Satisfaction with service facilities exerts a significant positive effect on cultural perception.

H5: nearby public cultural facilities perception partially mediates the relationship between satisfaction with interior spatial design and cultural perception.

H6: nearby public cultural facilities perception partially mediates the relationship between satisfaction with entrance and exit design and cultural perception.

H7: nearby public cultural facilities perception partially mediates the relationship between satisfaction with service facilities and cultural perception.

## Materials and methods

### Study design and analytical framework

**Fig 2:**
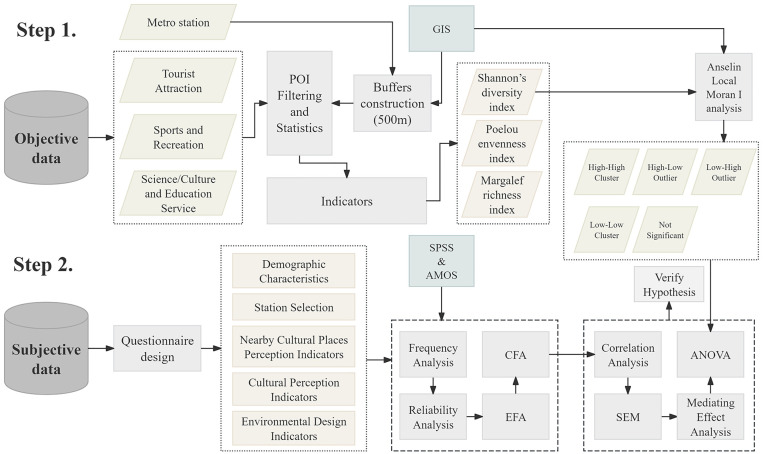
Analytical workflow of the study. Created by the authors.

To address the research questions, the study follows a two-stage workflow (Fig 2). Stage 1(objective data): 500 m circular buffers were built around candidate stations, and POI records were filtered and aggregated. The Shannon diversity index served as the primary metric for the diversity of public cultural facilities, with Pielou’s evenness and Margalef’s richness used as auxiliary diagnostics. Anselin Local Moran’s I (LMI) was then applied to identify four station types: High-High cluster (HH), High-Low outlier (HL), Low-High outlier (LH), and Low-Low cluster (LL). Hereafter, we refer to these as HH, HL, LH, and LL. Details are provided in Study area and Data sources and preprocessing. Stage 2(subjective data): an online questionnaire collected demographics, station choice, nearby public cultural facilities perception, cultural perception, and evaluations of interior spatial design, entrance and exit design, and service facilities. Statistical analyses included reliability testing, Exploratory factor analysis (EFA), Confirmatory factor analysis (CFA), Structural equation modeling (SEM) with bootstrapped tests for mediation, and one way ANOVA for differences across station types; methodological details appear in subsequent sections.

### Study area

**Fig 3:**
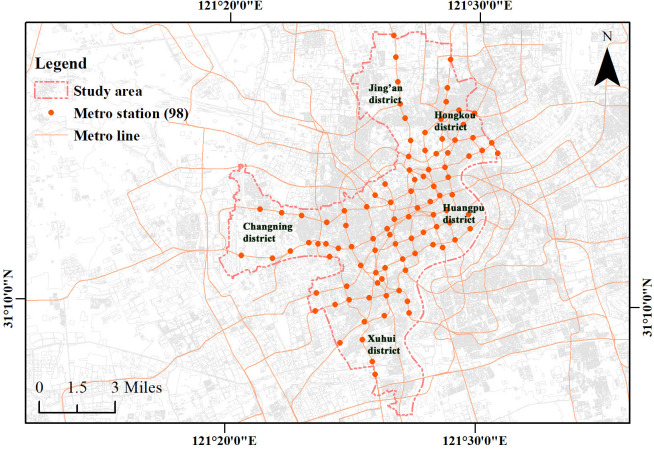
Study area. Base map and data from OpenStreetMap and OpenStreetMap Foundation. Visualization by the authors.

According to the 2024 Shanghai Statistical Yearbook [49], Shanghai consists 16 administrative districts and has 586 metro stations across on 20 lines. The urban core is defined as the area within the Outer Ring Road and includes five central districts (Huangpu, Xuhui, Changning, Jing’an, and Hongkou) with a total area of approximately 289.44 km2 and a resident population of 6.3833 million. Official data from the Shanghai Municipal Bureau of Planning and Natural Resources [50] indicate that 44 historic and cultural character conservation areas have been designated citywide, 11 of which are located within these five core districts, reflecting a high and representative concentration of cultural resources. Accordingly, these five districts were selected as the study area for station selection (Fig 3).

### Data sources and preprocessing

#### Objective data.

**Fig 4:**
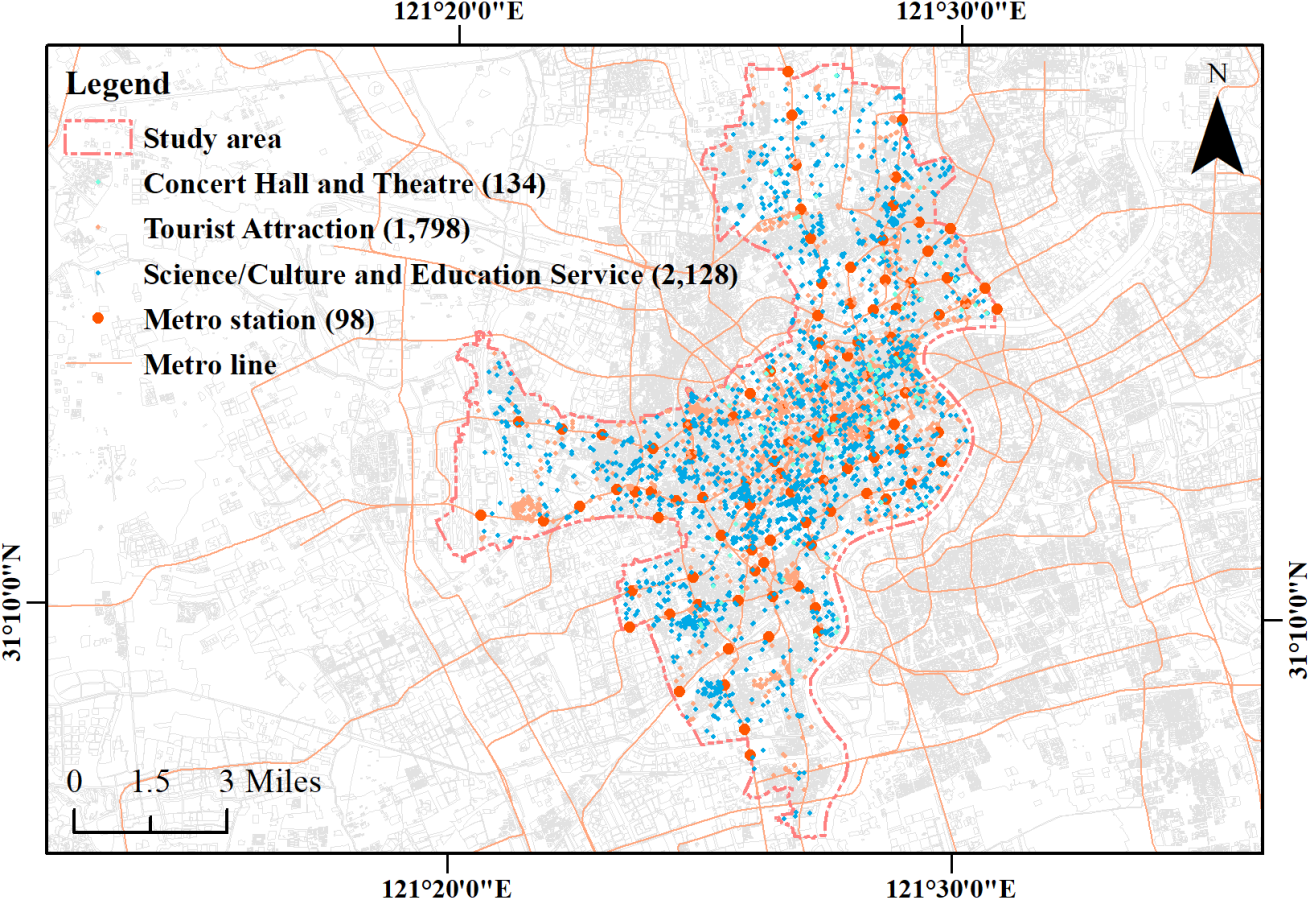
Spatial distribution of POI related to public cultural facilities in the study area. Base map and data from OpenStreetMap and OpenStreetMap Foundation. Visualization by the authors.

Administrative boundary data for Shanghai as of January 2025 and coordinates for 586 metro stations were obtained via the Amap Open Platform API. After deduplication and filtering to the study area, 98 candidate stations remained. We compiled 4,060 POI from 26 subcategories under the major classes of tourist attractions, science, education and culture services, and sports and leisure services to characterize the spatial distribution of public cultural facilities (Fig 4). All maps were rendered on an OpenStreetMap basemap, with attribution to OpenStreetMap contributors under the ODbL.

Given the high population and station densities in central Shanghai, and the common use of a 500 m walking radius in East Asian urban studies [54], the station service area was defined as a 500 m circular buffer. Buffers were generated in ArcGIS 10.8.1; for transfer stations, separate buffers were retained, and deduplication yielded 98 valid buffers in total.

**Fig 5:**
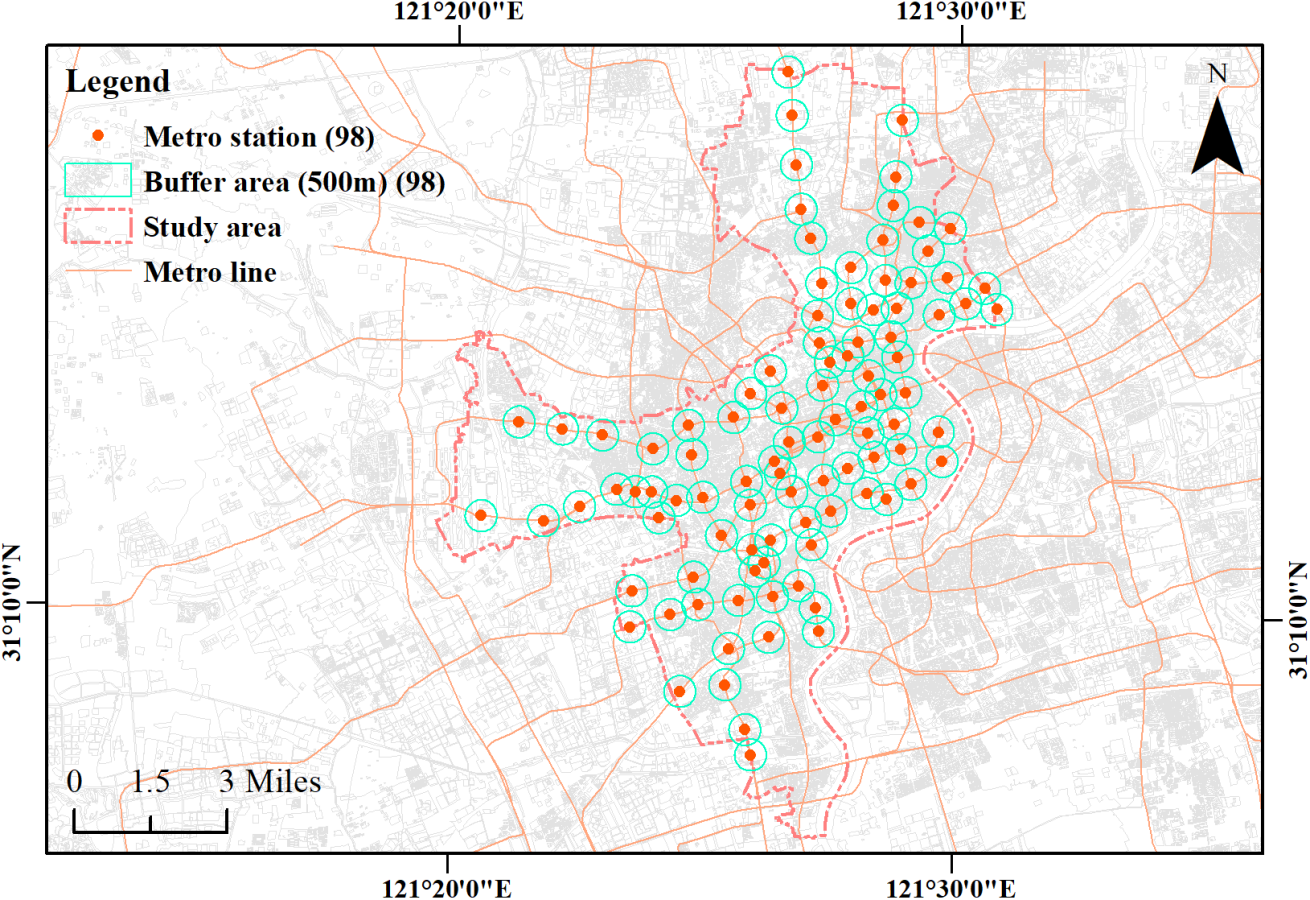
Distribution of public cultural facility-related POIs within buffers of stations in the study area. Base map and data from OpenStreetMap and OpenStreetMap Foundation. Visualization by the authors.

After removing duplicates and irrelevant records, a spatial overlay was performed for each 500 m buffer to count valid POI within the station service area, yielding 2,437 records in total (Table 1). All spatial analyses were conducted in the WGS_1984_UTM_Zone_51N projected coordinate system (Fig 5).

**Table 1:** Classification and statistics of the number of POIs within the study area.

Big Category	Sub Category	Number		Sum	
		500m buffer	five districts	500m buffer	five districts
**Tourist Attraction**	Park	122	213	1107	1798
	Park & Square	9	11		
	City Plaza	75	110		
	Scenery Spot	134	219		
	National View Spot	2	4		
	Provincial View Spot	1	4		
	Tourist Attraction	564	932		
	Red scenic spot	37	57		
	Memorial Hall	99	150		
	Church	19	33		
	Buddhist & Taoist Temple	44	63		
	Mosque	1	2		
**Science/Culture & Education Service**	Science & Education Cultural Place	535	932	1229	2128
	Museum	53	75		
	Exhibition Hall	87	126		
	Convention & Exhibition Center	40	86		
	Art Gallery	39	75		
	Library	61	99		
	Science & Technology Museum	4	8		
	Planetarium	0	2		
	Cultural Palace	46	80		
	Archives Hall	7	15		
	University & College	181	318		
**Sports & Recreation**	Research Institution	176	312	101	134
	Concert Hall	3	5		

#### Subjective data.

Subjective data were collected through an online structured questionnaire on the Credamo platform. Fieldwork ran from March 8 to March 25, 2025 and yielded 414 valid responses. Respondents were recruited by convenience sampling. The target population comprised Shanghai residents aged 18 years or older who ride the metro frequently. To constrain the sample and improve relevance, access to the questionnaire was restricted on the platform to users whose IP geolocation was in Shanghai. The first page contained a screening item asking whether the respondent regularly rides the metro; only those answering yes proceeded to the main survey. The instrument included a usual station item for type-based comparisons. Respondents selected their usual station from a pre-set list of twelve stations created by randomly drawing three representative stations from each of the four station types derived from the Shannon diversity index and Anselin Local Moran’s I. All respondents provided electronic informed consent online prior to participation (S1 Appendix).

Demographic variables were coded as follows: gender (male = 1, female = 2); age (five levels coded 1–5); education (three levels coded 1–3). The usual station variable was coded 1–12 and, during processing, tagged with its station-type label HH, HL, LH, or LL. All Likert items were aligned in direction with no reverse coded items and therefore required no reverse scoring. Data preprocessing followed predefined validity rules and excluded incomplete or logically inconsistent responses. The final sample counts by station were as follows: JiaShan Lu (n = 34), NanJingDong Lu (n = 44), YuYuan (n = 33), BeiXinJing (n = 36), CaoBao Lu (n = 36), YanChang Lu (n = 32), ZiRanBoWuGuan (n = 32), LaoXiMen (n = 33), LuJiaBang Lu (n = 32), LongHua (n = 31), ShiLong Lu (n = 33), and SiPing Lu (n = 38). The complete questionnaire data are provided in S2 Data.

#### Public cultural facility diversity indices.

The Shannon diversity index(H′) was originally developed in ecology to measure species diversity and, in essence, it captures the information entropy of a set of discrete categories; the more difficult it is to predict the type of a randomly drawn individual within a given spatial unit, the higher the index [52,53]. In urban studies, the index has been widely used to characterize the diversity of facility and function types within spatial units, as well as land use mix and complexity [54–56]. We classified and aggregated public cultural facilities using the POI classification codes of the AMap Open Platform [57], using its third-level categories to treat the different facility types within the 500 m station-area buffer (for example, museums, theaters, and art galleries) as categories and the counts of each type as individuals, and cross-checked the category mapping against the national standard GB/T 35648−2017 [58].

Additionally, we computed Pielou’s evenness (J′) and Margalef richness (R), which capture the balance across categories and the number of categories, respectively. Because Shannon diversity (H’) reflects both richness and evenness, it was used as the primary indicator in the subsequent clustering, with J′ and R serving as descriptive supplements. A higher H′ indicates that the types of facilities encountered within the buffer are harder to predict, implying a more diverse and more even supply. The formulas are as follows:


$$\begin{array}{c} H^{\prime }=-\sum {\mathrm{\backslash nolimits}}_{i=\text{1}}^{S}p_{i}\text{ ln}(p_{i}) \end{array}$$

**Equation 1** Shannon’s diversity index.

**Equation 2** Pielou’s evenness index.

**Equation 3** Margalef richness index.

Where H′ is the Shannon diversity index; S is the total number of nearby public cultural facilities types; P_i_ is the proportion of nearby public cultural facilities of type i within the station area (count of type i divided by N); J′ is Pielou’s evenness index; N is the total number of nearby public cultural facilities in the station area; R is Margalef’s richness index; and ln denotes the natural logarithm.

### Classification of metro stations based on local Moran’s I

Local clustering of public cultural facility type diversity was assessed with Anselin Local Moran’s I (ArcGIS 10.8.1). For each station, the Shannon diversity index (H′) was computed within a 500 m circular buffer. Spatial weights used a row-standardized inverse-distance-squared scheme within a 500 m distance band (self-weights excluded; locations beyond the band received zero weight). H′ values were standardized to z-scores, and the neighborhood mean of H′ was calculated using the same weights. Local Moran’s I statistics and pseudo p-values were obtained by permutation, with significance set at p < 0.05. Stations with significant results were classified as HH, HL, LH, or LL according to whether the station’s standardized H′ and its neighborhood mean were above or below the overall mean; all others were labeled not significant.

### Questionnaire design and measures

As detailed in Subjective data sources and preprocessing, data were collected via an online structured questionnaire on the Credamo platform; this section describes the instrument and measures.

The main questionnaire consisted of two parts. The first part collected demographics and travel information, namely gender, age, educational attainment, and usual station. The usual station list contained twelve options constructed by randomly selecting three representative stations from each of the four station types defined by the Shannon diversity index within the 500 m station area and Anselin Local Moran’s I. The typology was not disclosed to respondents, and an Other/Not listed option was provided.

The second part employed a five-point Likert scale with 21 items covering five dimensions: satisfaction with interior spatial design, satisfaction with entrance and exit design, satisfaction with service facilities, perceptions of nearby public cultural facilities, and overall cultural perception of the station. Items were adapted from prior studies and aligned to the study context. Indicator definitions are reported in Table 2, and the full instrument is provided in S3 Appendix. To ensure consistent interpretation, satisfaction items were coded from 1 (very dissatisfied) to 5 (very satisfied), and perception items from 1 (strongly disagree) to 5 (strongly agree). No reverse coded items were used.

**Table 2:** Measurement items and references.

Dimensional	Index	Item Number	Reference
**Station Design and Facilities Satisfaction**	Satisfaction with entrance and exit size	D1	[3,6,7,20,29,38,59]
	Satisfaction with entrance and exit decorative elements	D2	
	Satisfaction with entrance and exit sign design	D3	
	Satisfaction with entrance and exit public art	D4	
	Satisfaction with entrance and exit landscape design	D5	
	Satisfaction with interior public art	D10	
	Satisfaction with interior cultural activities	D11	
	Satisfaction with interior wall design	D12	
	Satisfaction with interior ceiling design	D13	
	Satisfaction with interior floor design	D14	
	Satisfaction with interior guide system	D15	
	Satisfaction with interior supporting facilities	D16	
	Satisfaction with interior advertising facilities	D17	
**Station area Public Cultural Facilities Perception**	Perceived sufficiency of nearby public cultural facilities	D6	[5,18,29,31,44,48,49]
	Perceived diversity of nearby public cultural facilities	D7	
	Perceived prominence of nearby public cultural facilities	D8	
	Perceived accessibility of nearby public cultural facilities	D9	
**Metro Station Cultural Perception**	Perceived cultural atmosphere	D18	[7,8,20,26–28]
	Perceived historical background	D19	
	Perceived design style	D20	
	Perceived regional characteristics	D21	

### Analytical framework based on SEM

SEM is widely used to characterize relations among latent factors in psychology and the social sciences [60,61]. The approach estimates the measurement model that links observed indicators to latent variables together with the structural model that specifies directional relations among latent variables, which enables identification of direct and indirect effects across multivariate constructs [62]. The workflow comprised EFA, CFA, and estimation of the structural model; routine checks on normality, descriptive statistics, reliability, and one-way analysis of variance were also conducted and are reported with the results.

Exploratory factor analysis was conducted in SPSS version 27 using principal components extraction with varimax rotation. Factors were retained when eigenvalues > 1.0 and cumulative explained variance ≥ 60%. Sampling adequacy was confirmed by KMO > 0.70 and Bartlett’s test of sphericity with P < 0.001. To reduce subjectivity, retention and revision thresholds were prespecified: standardized loadings ≥ 0.50; where cross loadings occurred, secondary loadings < 0.30 and a difference between primary and secondary loadings ≥ 0.20; communalities ≥ 0.30. Items showing extreme skewness or kurtosis, semantic redundancy, or ambiguity were considered for revision or removal. The purpose of EFA was to identify latent dimensions rather than to perform mere dimensionality reduction [63,64].

After EFA confirmed the dimensional structure, the measurement model was specified on theoretical grounds and item content and was estimated in AMOS version 28. Model fit was judged by CMIN/DF < 3, RMSEA < 0.08, CFI ≥ 0.90, TLI ≥ 0.90, and SRMR < 0.08. Convergent validity required (CR) ≥ 0.70 and average variance extracted (AVE) ≥ 0.50. Limited modifications, such as combining items or freeing error covariances, were considered only when theoretically justified.

With the measurement model established, the structural model set the environment related dimensions (interior spatial design satisfaction, entrance and exit design satisfaction, service facilities satisfaction) as exogenous latent variables, nearby public cultural facilities perception as a mediating latent variable, and the station’s overall cultural perception as the endogenous latent variable. Maximum likelihood estimation produced standardized path coefficients, and statistical significance was evaluated at P < 0.05. Structural model fit used the same criteria listed above. Mediation was tested with bootstrap resampling of B = 2000 and bias corrected 95% confidence intervals; mediation was deemed significant when the interval excluded zero. When appropriate, the variance accounted for (VAF), was reported to aid interpretation of indirect effect size [65].

### Ethics statement

This study used a non-interventional, minimal risk, anonymous online survey and collected no sensitive content or personally identifiable information. The study protocol, including the design, recruitment procedures, and data handling, complied with the Administrative Measures for the Ethics Review of Science and Technology at Jingdezhen Ceramic University and with the Measures for the Ethical Review of Human Related Scientific Research (Trial) (MOST Supervision [2023] No. 167). Participation was voluntary after electronic informed consent on the Credamo platform, and only individuals 18 years or older were eligible. The exported and analysis datasets exclude names, contact information, precise geolocation, IP addresses, device identifiers, and other potential identifiers; platform technical logs were not downloaded or analyzed. Data were used solely for academic purposes and were stored and processed in anonymized form with restricted access. The study adhered to the Declaration of Helsinki and applicable data protection and privacy regulations.

## Results

### Public cultural facility diversity and metro station classification

Based on POI data within 500 m buffer zones surrounding 98 metro stations in the study area, 90 valid stations were obtained for analysis. Descriptive statistics revealed substantial spatial heterogeneity in cultural diversity indicators across these stations (S4 Data). The number of nearby public cultural facilities types (S) ranged from 2 to 16, reflecting a differentiated distribution of public cultural facility types. H′ spanned from 0.637 to 2.405, indicating large variation in both richness and evenness across stations. J′ ranged from 0.421 to 1.000, with values closer to 1 indicating more even type distributions and lower values indicating dominance by a few types. R ranged from 0.721 to 3.687, consistent with variation in the scale of cultural resources across urban functional zones.

**Fig 6:**
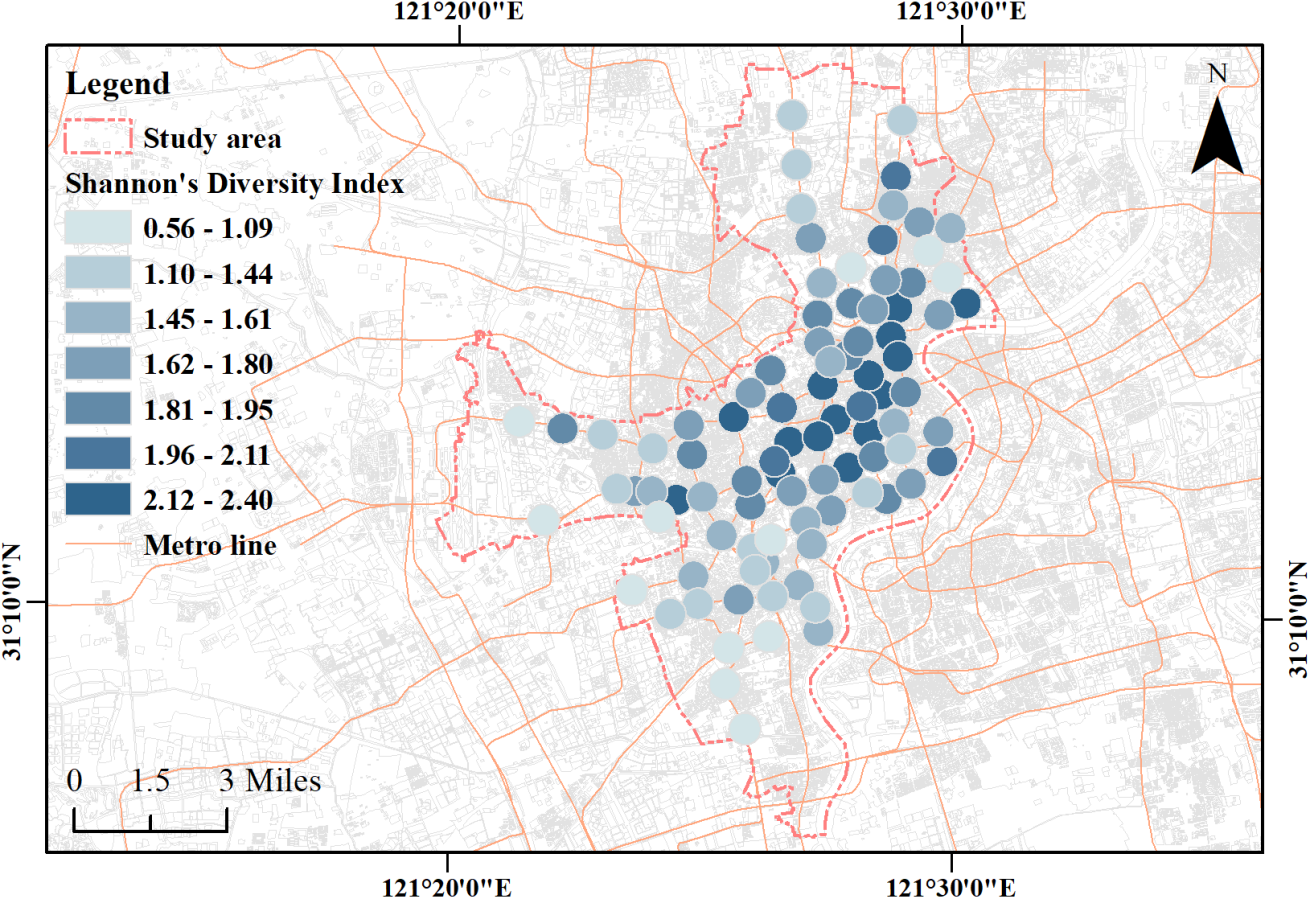
Shannon diversity index across 500 m station buffer zones. Base map and data from OpenStreetMap and OpenStreetMap Foundation. Visualization by the authors.

To visualize cultural diversity across stations, the H′ was mapped in ArcGIS using Jenks natural breaks, dividing stations into seven levels (Fig 6). The spatial pattern revealed a core to periphery gradient, with high diversity stations clustered in central urban areas and lower diversity stations increasingly concentrated near administrative boundaries. This pattern corresponded closely with the counts of nearby public cultural facilities related POI shown in Fig 3.

**Fig 7:**
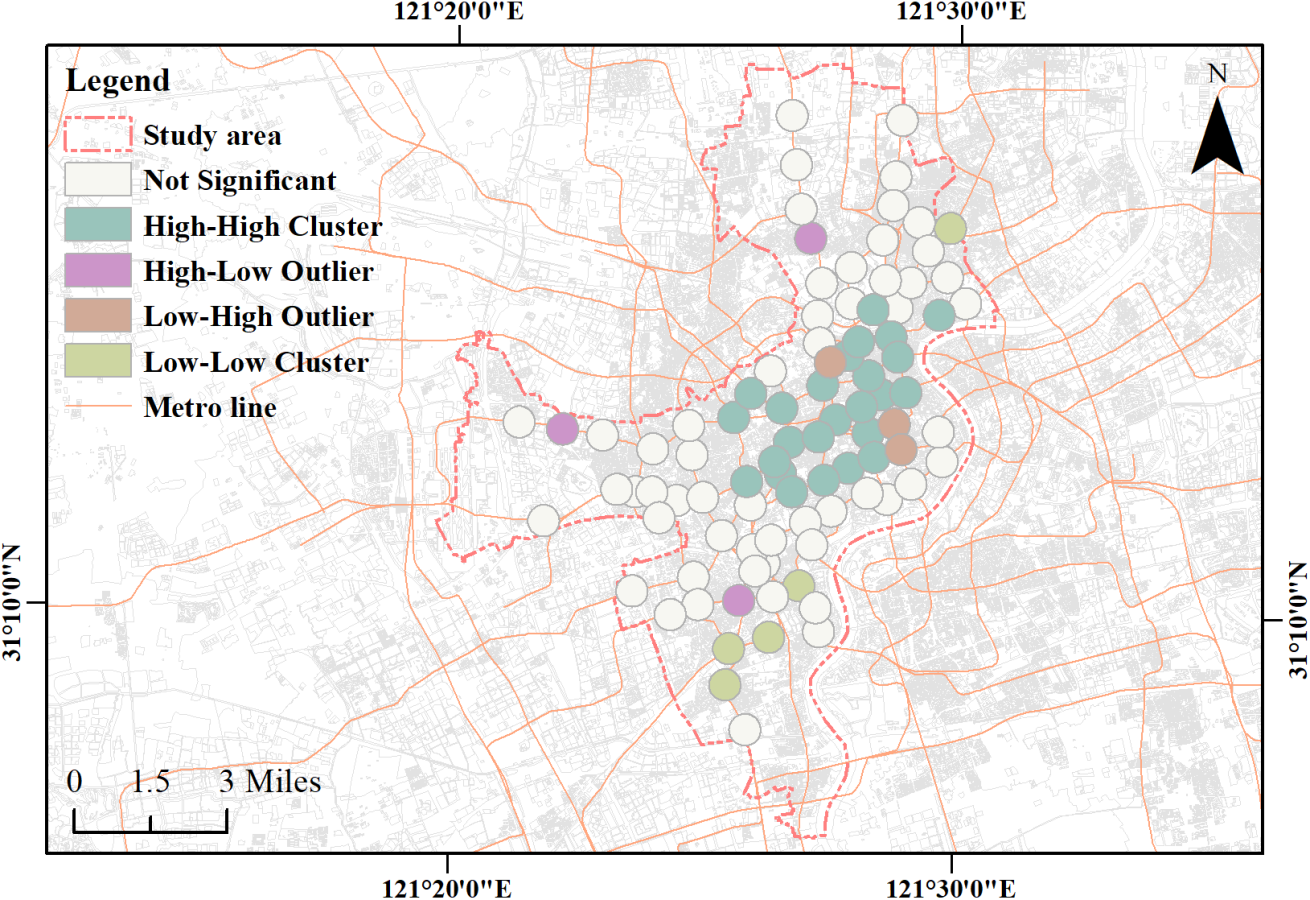
LMI clustering of Shannon diversity index. Base map and data from OpenStreetMap and OpenStreetMap Foundation. Visualization by the authors.

Using station-level H′ as the “value” variable, LMI identified four types (Fig 7). Here, “high/low-value area” simply refers to higher/lower H′ at the focal station and, for context, the neighborhood average of H′ among its LMI neighbors. Accordingly:

HH (N = 25; H′ = 1.667–2.405; neighborhood mean of H′ = 1.828–2.181): stations with high H′ and a high neighborhood mean of H′.
HL (N = 3; H′ = 1.681–1.927; neighborhood mean of H′ = 1.171–1.343): stations with high H′ but a low neighborhood mean of H′.
LH (N = 3; H′ = 1.409–1.606; neighborhood mean of H′ = 1.866–1.951): stations with low H′ but a high neighborhood mean of H′.
LL (N = 5; H′ = 0.950–1.602; neighborhood mean of H′ = 0.950–1.201): stations with low H′ and a low neighborhood mean of H′.

All cluster assignments were statistically significant under LMI (Z scores and p values) (Table 3), supporting the statistical validity of the clustering results. Details for all stations are provided in S5 Data.

**Table 3:** Ranges of LMI statistics for clustered metro stations.

CO Type	H’	LMi Index	LMi Z-Score	LMi P-Value	N Neighbors	Neighborhood means of H′
**HH**	[1.667, 2.405]	[0, 0.016]	[1.660, 3.699]	[0.002, 0.046]	[4, 14]	[1.828, 2.181]
**HL**	[1.681, 1.927]	[-0.001, 0]	[-2.347, -1.777]	[0.014, 0.050]	[2, 9]	[1.171, 1.343]
**LH**	[1.409, 1.606]	[-0.003, -0.001]	[-2.260, -1.921]	[0.006, 0.024]	[11, 12]	[1.866, 1.951]
**LL**	[0.950, 1.602]	[0.001, 0.005]	[1.680,2.740]	[0.006, 0.044]	[2, 10]	[0.950, 1.201]

### Sample characteristics

Descriptive statistics of the demographic characteristics from the 414 valid survey responses are presented in Table 4. Gender distribution was approximately balanced, and most participants were between 20–40 years old, which corresponds to the primary commuting demographic of metro passengers. Most respondents reported higher educational attainment, and sample sizes across the twelve selected stations were relatively even, providing broad coverage across the selected sites.

**Table 4:** Descriptive statistics of demographic variables.

Demographic Item	Category	Frequency	Percentage
**Gender**	Male	204	49.30%
	Female	210	50.70%
**Age**	Under 20 years old	33	8%
	20-30 years old	215	51.90%
	31-40 years old	98	23.70%
	41-50 years old	35	8.50%
	Over 50 years old	33	8%
**Education Level**	High school degree or below	44	10.60%
	College/Bachelor degree	249	60.10%
	Postgraduate degrees	121	29.20%

The distribution of respondents across the four station types is shown in Table 5, with the proportions being 26.81% (HH), 25.12% (HL), 23.43% (LH), and 24.64% (LL), respectively. The sample distribution across station types was thus relatively balanced, and each station had at least 30 valid responses, meeting common recommendations for subsequent statistical analysis.

**Table 5:** Sample distribution and proportions by station and station type.

Station name	Frequency	Percentage	Station type
JiaShan Lu	34	8.21%	HH
NanJingDong Lu	44	10.63%	
YuYuan	33	7.97%	
BeiXinJing	36	8.70%	HL
CaoBao Lu	36	8.70%	
YanChang Lu	32	7.73%	
ZiRanBoWuGuan	32	7.73%	LH
LaoXiMen	33	7.97%	
LuJiaBang Lu	32	7.73%	
LongHua	31	7.49%	LL
ShiLong Lu	33	7.97%	
SiPing Lu	38	9.18%	

Reliability analysis using SPSS version 27 showed that the overall Cronbach’s alpha (α) of the 414 questionnaires was 0.952. The environment related evaluation and cultural perception subscales yielded α = 0.941 and α = 0.889, respectively. All values were ≥ 0.80, confirming high internal consistency of the collected data and supporting their suitability for further analysis.

### Measurement model: EFA and CFA

#### Exploratory factor analysis (EFA).

Principal component analysis (PCA) with varimax (orthogonal) rotation was conducted in SPSS version 7 on 17 built environment related items. Sampling adequacy was high (KMO = 0.934), and Bartlett’s test supported factorability (p < 0.001) Retaining factors with eigenvalues > 1.0 and standardized loadings ≥ 0.50 yielded four factors accounting for 73.371% of the total variance. The solution aligned with theory: F1 = interior spatial design satisfaction; F2 = nearby public cultural facilities perception; F3 = service facilities satisfaction; and F4 = entrance and exit design satisfaction. No items showed low primary loadings or salient cross loadings. The dependent variable (cultural perception) was not included in this EFA. Detailed loadings and variance shares appear in Table 6.

**Table 6:** Results of exploratory factor analysis.

Items	Common Factor			
	F1	F2	F3	F4
**Cumulative variance contribution rate**	22.50%	40.77%	58.00%	73.37%
**Initial eigenvalue**	8.171	1.888	1.267	1.146
**Explain variance**	22.50%	18.27%	17.23%	15.38%
**Variable item (factor load)**	D11 0.800	D7 0.846	D15 0.788	D5 0.777
	D10 0.789	D6 0.833	D16 0.787	D1 0.760
	D13 0.787	D8 0.787	D17 0.727	D4 0.758
	D12 0.783	D9 0.744	D3 0.725	D2 0.538
	D14 0.783			
**Mean value**	3.398	3.567	3.813	3.807

D1–D17 are observed indicators. Factor loadings reports rotated standardized loadings (the highest loading for each item is shown). Explained variance is the percent explained by each factor; “cumulative variance explained” is the running total. Mean is the average item score on a five-point scale.

#### Confirmatory Factor Analysis (CFA).

**Fig 8:**
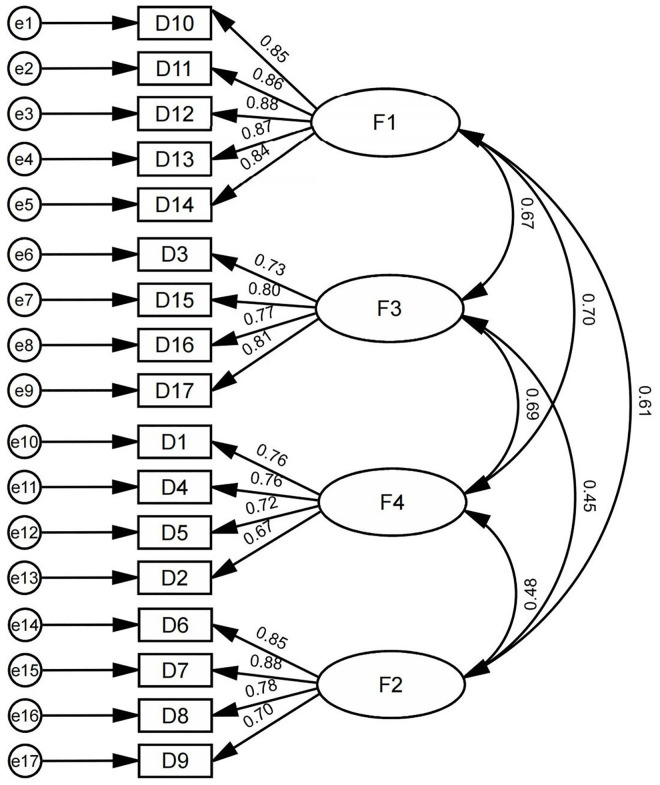
Measurement model. Created by the authors. F1 = interior spatial design satisfaction; F2 = perceptions of nearby public cultural facilities; F3 = service facilities satisfaction; F4 = entrance and exit design satisfaction.

CFA was estimated in AMOS version 28 to evaluate the measurement model relating four sets of indicators to their target latent constructs. Overall fit was satisfactory: CMIN/DF = 2.741 (within the commonly accepted range of 1–3) and RMSEA = 0.065 (< 0.08). Incremental and comparative indices were likewise high (NFI = 0.934, RFI = 0.921, IFI = 0.957, TLI = 0.948, CFI = 0.957) each ≥ 0.90, indicating a well-fitting model. Collectively, the results support strong construct validity for the CFA specification of environmental design factors. Latent constructs exhibited moderate-to-strong positive intercorrelations, and all standardized factor loadings were ≥ 0.60, evidencing adequate indicator reliability. Standardized paths for the measurement model are presented in Fig 8.

According to the results shown in Table 7, the AVE for each dimension exceeded 0.50, and the CR for each exceeded 0.80, demonstrating that each dimension possesses good convergent validity and composite reliability.

**Table 7:** Measurement model results: CFA, CR, and AVE values.

Path			Estimate	AVE	CR
D14	←	F1	0.836	0.74	0.934
D13	←	F1	0.873		
D12	←	F1	0.883		
D11	←	F1	0.855		
D10	←	F1	0.853		
D9	←	F2	0.703	0.649	0.88
D8	←	F2	0.782		
D7	←	F2	0.878		
D6	←	F2	0.848		
D17	←	F3	0.808	0.602	0.858
D16	←	F3	0.768		
D15	←	F3	0.796		
D3	←	F3	0.728		
D2	←	F4	0.667	0.529	0.818
D5	←	F4	0.719		
D4	←	F4	0.764		
D1	←	F4	0.756		

D1–D17 denote observed indicators, F1–F4 denote latent constructs. Estimate reports standardized CFA loadings from indicator Di to construct Fj. AVE denotes average variance extracted; CR denotes composite reliability. For each construct, AVE and CR are shown in the first row of its block.

In addition, according to the results shown in Table 8, the square root of each dimension’s AVE exceeded that dimension’s correlations with the other latent variables, indicating good discriminant validity among the four latent dimensions and supporting the established measurement model for use in subsequent structural analysis.

**Table 8:** Comparison of square roots of AVE and inter factor correlations.

Factor	F1	F2	F3	F4
**F1**	**0.74**			
**F2**	0.614	**0.649**		
**F3**	0.667	0.447	**0.602**	
**F4**	0.699	0.484	0.694	**0.529**
**Square root of the AVE**	0.86	0.806	0.776	0.728

F1 = interior spatial design satisfaction; F2 = perceptions of nearby public cultural facilities; F3 = service facilities satisfaction; F4 = entrance and exit design satisfaction.

### Descriptive statistics, normality, and correlations

As the statistical methods employed in this study require the data to follow a normal distribution, normality tests were conducted prior to analysis. As shown in Table 9, mean scores at both the dimension and item levels ranged from 3.0 to 4.0 on a five-point Likert scale (higher values indicate more positive evaluations), indicating generally positive responses across all five dimensions.

**Table 9:** Normality test results of scale variables.

Dimension	Item	AVE	SD	Skewness	Kurtosis	D_AVE	D_SD
**F1**	D10	3.24	0.9	0.161	−0.822	3.398	0.784
	D11	3.26	0.895	0.064	−0.898		
	D12	3.5	0.896	−0.081	−0.75		
	D13	3.46	0.906	−0.054	−0.796		
	D14	3.53	0.81	−0.075	−0.477		
**F2**	D6	3.47	0.963	0.145	−0.935	3.567	0.81
	D7	3.49	0.948	0.055	−0.907		
	D8	3.55	0.96	0.002	−0.952		
	D9	3.76	0.909	−0.151	−0.871		
**F3**	D3	3.91	0.681	−0.066	−0.394	3.813	0.615
	D15	3.82	0.738	−0.249	−0.16		
	D16	3.78	0.751	−0.339	−0.053		
	D17	3.74	0.767	−0.273	−0.202		
**F4**	D1	3.89	0.676	−0.334	0.279	3.807	0.568
	D4	3.74	0.702	−0.1	−0.215		
	D5	3.79	0.695	−0.178	−0.1		
	D2	3.8	0.764	−0.144	−0.425		
**F5**	D18	3.54	0.842	−0.02	−0.584	3.566	0.735
	D19	3.5	0.857	0.101	−0.632		
	D20	3.57	0.84	−0.053	−0.576		
	D21	3.64	0.853	0.07	−0.742		

F1 = interior spatial design satisfaction; F2 = perception of nearby public cultural facilities; F3 = service facilities satisfaction; F4 = entrance and exit design satisfaction.

F5 = culture perception

The normality of each measurement item was assessed using skewness and kurtosis statistics. According to the criteria proposed by Kline [63], absolute skewness values < 3 and absolute kurtosis values < 8 were taken as acceptable indicators of approximate normality. All items met these thresholds, supporting the suitability of the data for further analysis.

Pearson correlation analysis was performed to examine the relationships among the variables. Results shown in Table 10 show that all coefficients were > 0 and statistically significant at p < 0.01 (two tailed), indicating positive and significant associations and supporting the appropriateness of the data for subsequent modeling.

**Table 10:** Inter-variable relationships tested using Pearson correlation.

Factor	F1	F2	F3	F4	F5
**F1**	1				
**F2**	.569**	1			
**F3**	.594**	.417**	1		
**F4**	.624**	.435**	.588**	1	
**F5**	.730**	.572**	.518**	.591**	1

### Structural model results for direct and indirect effects

A structural equation model was estimated in AMOS version 28 to evaluate the relationships between four independent variables (interior spatial design satisfaction, nearby public cultural facilities perception, service facilities satisfaction, entrance and exit design satisfaction) and one dependent variable (cultural perception). Model fit indices demonstrated satisfactory results: CMIN/DF (χ²/df) = 2.326, within the acceptable range of 1–3; RMSEA = 0.057, below the 0.08 threshold; SRMR = 0.046, below the 0.05 benchmark. Other fit indices were also strong: NFI = 0.932, RFI = 0.920, IFI = 0.960, TLI = 0.953, and CFI = 0.960 (each ≥ 0.90). These results indicate that the cultural perception SEM model demonstrates good model fit.

**Fig 9:**
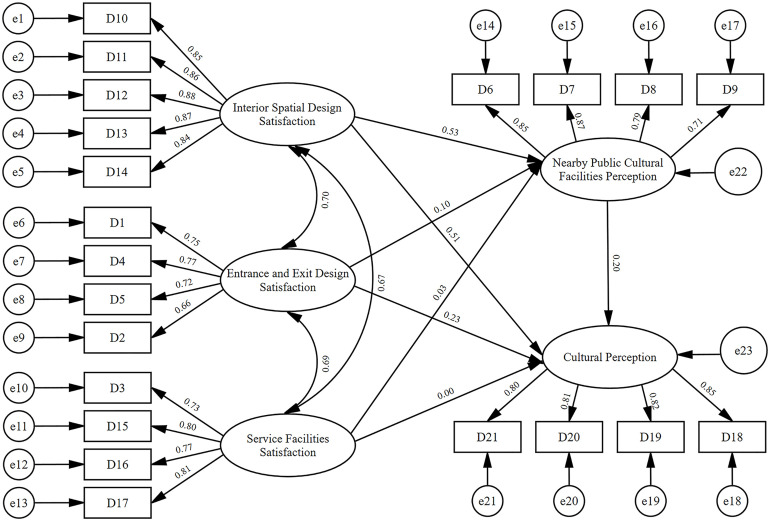
SEM of cultural perception and environmental design satisfaction. Created by the authors.

As illustrated in Fig 9, the hypothesis testing results show that: Perception of surrounding public cultural facilities significantly and positively predicted overall metro station cultural perception (β = 0.196, p < 0.001), supporting Hypothesis H2. Satisfaction with service facilities did not significantly predict cultural perception (β = 0.07, p > 0.05), hence Hypothesis H3 was not supported. Satisfaction with interior spatial design had a significant positive effect on cultural perception (β = 0.514, p < 0.001), supporting Hypothesis H1. Satisfaction with entrance and exit design also significantly and positively predicted cultural perception (β = 0.231, p < 0.001), confirming Hypothesis H4.

As shown in Table 11, the mediation effect tests revealed the following:

**Table 11:** Bootstrapped mediation test results with confidence intervals.

Path	Effect	Effect value	Bias-corrected 95% CI		Results
			Lower	Upper	
**F1 → F2 → F5**	Total effect	0.617	0.492	0.735	Significant partial mediation exists
	Direct effect	0.514	0.378	0.651	
	Indirect effect	0.103	0.042	0.187	
**F4 → F2 → F5**	Total effect	0.25	0.104	0.388	Only the direct effect is significant
	Direct effect	0.231	0.086	0.359	
	Indirect effect	0.019	−0.012	0.061	
**F3 → F2 → F5**	Total effect	0.008	−0.11	0.133	No statistically meaningful effects were detected
	Direct effect	0.002	−0.112	0.124	
	Indirect effect	0.006	−0.024	0.049	

Bootstrap resamples B = 2000, Bias-corrected confidence intervals, F1 = interior spatial design satisfaction; F2 = perceptions of nearby public cultural facilities; F3 = service facilities satisfaction; F4 = entrance and exit design satisfaction; F5 = cultural perception. Total effect = direct effect + indirect effect.

In Path 1 (Interior spatial design satisfaction → Perception of public cultural facilities → Cultural perception): both the total effect and the direct effect were significant. Additionally, the indirect effect via public cultural facilities perception was also significant, indicating a partial mediation effect. Thus, Hypothesis H5 is supported.

In Path 2 (Entrance and exit design → Perception of public cultural facilities → Cultural perception): the total and direct effects were significant, but the indirect effect was not. Therefore, public cultural facility perception did not mediate the relationship, and Hypothesis H6 is not supported.

In Path 3 (Service facilities satisfaction → Perception of public cultural facilities → Cultural perception): the total effect was not significant, indicating no mediation, and Hypothesis H7 is not supported.

### Group differences by one-way ANOVA

As shown in Table 12, a one-way ANOVA was conducted to examine differences among the four station types (HH, HL, LH, LL) in interior spatial design satisfaction, cultural perception, perception of nearby public cultural facilities, and entrance and exit design satisfaction.

**Table 12:** ANOVA results for different station types.

Factor	Each dimension (mean ± standard deviation)				F	p
	HH(n = 111)	HL(n = 104)	LH(n = 97)	LL(n = 102)		
**F1**	3.73 ± 0.75	3.53 ± 0.73	3.57 ± 0.71	1.00 ± 0.00	437.234	0.000**
**F2**	4.12 ± 0.74	3.63 ± 0.78	3.50 ± 0.72	2.97 ± 0.52	48.894	0.000**
**F3**	3.92 ± 0.60	3.94 ± 0.59	3.78 ± 0.54	3.58 ± 0.45	9.574	0.000**
**F5**	3.91 ± 0.65	3.75 ± 0.74	3.50 ± 0.72	3.07 ± 0.53	31.211	0.000**

* p < 0.05 ** p < 0.01.

The results indicate that significant differences were found across all four variables (p < 0.01), with the following specific findings:

Interior spatial design satisfaction: The differences among groups were highly significant (F = 437.234, p < 0.001). Mean score ordering: HH > HL > LL, and LH > LL.

Cultural Perception: Significant differences were observed (F = 31.211, p < 0.001). Mean score ordering: HH > LH/LL, HL > LH/LL, and LH > LL.

Perception of nearby public cultural facilities: The differences were also significant (F = 48.894, p < 0.001). Mean score ordering: HH > HL/LH/LL, and HL/LH > LL.

Entrance and exit design satisfaction: A significant group effect was found (F = 9.574, p < 0.001). Mean score ordering: HH/HL/LH > LL.

## Discussion

### Determinants and pathways of cultural perception

According to the results, interior spatial design, entrance and exit design, and the perception of nearby public cultural facilities all show significant positive effects on cultural perception. This conclusion is consistent with studies focusing on station cultural image and cultural landscape perception, indicating that improvements in environmental and spatial design can enhance passengers’ cultural image perception [8,34]. Although some studies model “environmental design” as a second-order latent variable while the present study treats design elements as first-order variables, both exhibit consistency on the main path from design cues to cultural perception [34]. This is partly aligned with the SOR proposition in environmental psychology, namely that physical cues as stimuli are internalized into psychological representations that influence overall evaluation and behavior [66,67].

In terms of effect size, interior spatial design plays a more prominent role. The reason is that interiors more readily generate high-salience legible and imageable cues—such as public art, materials and colors, lighting, and spatial organization—which are more likely to be noticed, encoded in memory, and incorporated into evaluation [23,26]. Entrances and exits must align clearly with surface destinations; although design freedom is relatively constrained, their recognizability and directional clarity still contribute steadily to overall judgments [16,17]. By contrast, wayfinding, auxiliary, and advertising facilities are typically used briefly on demand and are highly standardized, making their salience lower in attentional competition and their independent incremental effects limited; this accords with evidence that effective wayfinding primarily improves wayfinding performance rather than conferring cultural meaning [6,68]. These results are not entirely consistent with the traditional services cape generalization that the overall environment broadly shapes experience; the difference can be explained by context and scale, as functional cues are less likely to be individually transformed into cultural meaning within highly standardized metro systems at the station-area scale. It is also noteworthy that heritage and place-identity studies suggest that appropriate local expression supports differentiated cognition [31,35]; when service facilities are localized or themed, their influence may be underestimated by the present measurement model.

Mediation tests show that the perception of nearby public cultural facilities partially mediates the relationship between interior spatial design and cultural perception, and this effect appears primarily along the interior pathway. This finding is consistent with the node–place and TOD literature, which posits that station attributes must work in concert with station-area supply and continuity for passengers to obtain and translate exogenous cultural information [51,69]. When in-station wayfinding and information displays are clearly integrated at interior and entrance/exit interfaces and effectively link to nearby public cultural facilities such as museums and libraries, these cues are more readily incorporated into overall evaluations and strengthen cultural perception [6,31]. The mechanism is that interiors enhance legibility and imageability, providing a visual and locational frame for what exists, where it is, and how long it takes to reach, thereby reducing wayfinding and integration costs and incorporating external cultural anchors into cognitive maps and evaluative processes [68,70,71]. Based on this, we propose a testable proposition: when the types, prominence, quantity, and accessibility of public cultural facilities within the station area are more pronounced, and when information consistency and cross-station continuity are higher, the mediation effect may be stronger; when connectivity and consistency are weaker, evaluations are more likely to revert to the direct effects of interiors and entrances/exits.

### Station type differences

A one-way ANOVA shows that the four station types differ significantly in satisfaction with interior spatial design, satisfaction with entrance and exit design, perception of nearby public cultural facilities, and cultural perception. Given that the typology is derived from the Shannon diversity index of public cultural facilities within a 500 m service radius of the focal station and spatial clustering via LISA, these differences can be understood as the externalization of heterogeneity in station-area cultural supply in passengers’ subjective evaluations [43,57].

In the HH type, the categories within the focal station’s service area are rich and relatively contiguous with surrounding high-value patches. Repeated exposure within a consistent context facilitates a stable place representation, which tends to elevate both the perception of nearby public cultural facilities and overall cultural perception. At the same time, interior interfaces and entrances/exits are more easily embedded into a coherent urban narrative, raising their satisfaction ratings; this node–place synergy accords with classic perspectives that interpret station–area relations as complementary functions [51]. From the lens of place theory, a dense network of cultural anchors further consolidates sense of place and identity, thereby improving overall evaluations [70].

In the HL type, the key distinction is “strong supply at the focal station with weak support nearby.” Perception of nearby public cultural facilities is primarily determined by the quantity, types, and perceived accessibility within the focal station’s 500 m radius, so this dimension remains high. Concurrently, salient interior cues and clear entrance–surface alignment focus attention and memory on the focal station’s cues, lending resilience to interior and entrance/exit satisfaction through legibility [12,16]. Weakness at adjacent stations does not necessarily depress overall cultural perception, because evaluation benchmarks are anchored more to the focal station’s supply and interface quality.

In the LH type, insufficient supply at the focal station constrains perception of nearby public cultural facilities as measured within the 500 m radius, thereby suppressing overall cultural perception. Unlike HL, the advantage lies at adjacent stations rather than the focal one. Such external advantages do not change the definition or scope of the variables measured in this study, but they may function as contextual information that shapes passengers’ regional expectations and comparative judgments, thereby placing greater weight on direct evaluations of interiors and entrances/exits.

In the LL type, the category sets are narrow and lack continuity, making it difficult for passengers to capture external cues sufficient to sustain a place narrative during entry, dwell, and egress. Consequently, both perception of nearby public cultural facilities and overall cultural perception tend to be low. In this case, evaluations of interiors and entrances/exits rely more on baseline clarity and overall legibility, and the experience is more prone to homogenization, carrying a risk of placelessness [12,71].

### Design implications

Building on the above discussion, the following four points can guide design practice.

Use the station interior as an information carrier and systematically present the three dimensions of nearby public cultural facilities: count and type, recognizability, and perceived accessibility. Ensure that passengers can access this information during entry, waiting, and egress so that the interior contributes both directly to cultural perception and indirectly via nearby public cultural facilities.

Treat entrances and exits as a stable baseline. Strengthen clarity in scale, orientation, and interface so that they align clearly with surface destinations. Within regulatory standards, retain necessary local expression to avoid over standardization and loss of place-specific character.

Improve cross-station information continuity. Unify category coding and modes of expression for nearby public cultural facilities and ensure consistent presentation of related information on wayfinding and maps at adjacent stations. By station type, HH should emphasize information order and category clarity; HL and LH should reduce breaks and inconsistencies; LL should first supply basic category cues and access information.

Prioritize low-intervention and reusable implementation paths, including layered presentation, reuse of existing media, and incremental updates. Incorporate type-sensitive adjustments into routine maintenance to improve legibility and usability without adding new systems or maintenance burdens.

### Limitations and future research

This study has several limitations. First, both sampling and station type identification are confined to a single city, and the station area is defined by a 500 m radius, which constrains external validity. Second, the key variables are self-reported perceptions and the data come from an online repeated cross-sectional survey, so the estimates capture associations rather than strict causal effects. Future work could triangulate with offline surveys and objective behavioral data, such as opening hours of public cultural facilities and metro stations, ridership, and actual travel times, and employ longitudinal or quasi experimental designs to strengthen causal identification and external validity. We also recommend robustness checks for temporal heterogeneity, including survey date, weekday versus weekend, and peak versus off peak periods.

## Conclusion

This study was conducted at the 500 m station area scale and used metro stations in central Shanghai as a case study. We integrated spatial clustering based on Shannon diversity and local spatial autocorrelation with structural equation modeling to delineate pathways from the built environment of metro stations to cultural perception and to evaluate heterogeneity across station types. The results show that interior spatial design has the strongest association with cultural perception, followed by entrance and exit design. Perception of nearby public cultural facilities has an independent effect and partially mediates the association between interior spatial design and cultural perception. Station types derived from local spatial autocorrelation of Shannon diversity exhibit consistent differences in these outcomes. Therefore, uniform retrofit schemes are unlikely to be appropriate. Accordingly, the main conclusions are as follows.

(1) Interior spatial design is the strongest contributor to cultural perception, reflecting passengers’ evaluations of interior art, cultural programming, and surface design.
(2) Entrance and exit design is a secondary contributor, reflecting evaluations of entrance scale, decorative elements, public art, and landscape features.
(3) Perception of nearby public cultural facilities is positively associated with cultural perception and partially mediates the interior pathway, indicates that external cultural resources must work in concert with interior design to translate into higher cultural perception.
(4) Differences among station types are mainly related to the diversity and balance of cultural resources in the surrounding area. Environments with higher and more even diversity are more likely to achieve higher levels of cultural perception.

Overall, this study incorporates the subjective perception of the external cultural environment into the research framework for cultural perception in metro stations, clarifies the relationship between direct and mediated effects, and uses type differences to show the limitations of uniform strategies. The results provide a basis for factor prioritization and type stratification in station upgrades and retrofits and support more targeted design and operational strategies across different network contexts.

## Supporting information

S1 Appendix Electronic informed consent form. (DOCX)

S2 Data Dataset. Questionnaire raw data.
(XLS)

S3 Appendix Full survey instrument items and response scales. (DOCX)

S4 Data Dataset. Diversity and distribution indices of public cultural facilities within the 500 m buffers of 90 metro stations in central Shanghai.
(XLS)

S5 Data Dataset. Local spatial autocorrelation (LISA) results for Shannon diversity across metro station areas.
(XLS)
