# Supplementary material for: Identifying and modeling built environment factors influencing cultural perception in metro stations: Evidence from central Shanghai
Source: PLoS One. 2025 Nov 6;20(11):e0334642. doi: 10.1371/journal.pone.0334642 (PMC12591495; doi:10.1371/journal.pone.0334642)
Supplement: S1 Appendix — (DOCX) [file pone.0334642.s001.docx]

**Informed Consent Form**

Thank you for taking part in this study. To ensure accuracy and voluntary participation, please read and agree to the following information before you begin.

1. Research Unit, Project Team, and Contact

Research unit & project team: School of Design and Art, Jingdezhen Ceramic University

Principal Investigator (PI): Haoxuan Feng

Contact: Haoxuan Feng (2310012026@jcu.edu.com)

2. Study Content

This questionnaire aims to understand your perceptions of metro-station interior spatial design, entrance/exit design, service amenities, and nearby public cultural facilities (e.g., museums, libraries, cultural centers, art galleries). The goal is to examine how station-area built environment factors influence passengers’ cultural perception. Data will be used solely for academic research and statistical analysis, with no commercial use.

3. Estimated Duration

Approximately 6–10 minutes.

4. Data Confidentiality

This survey does not collect personally identifiable information (e.g., name, phone number, ID).

All data will be analyzed and reported in anonymous, aggregated form, stored on controlled devices/cloud services, and accessible only to the research team.

Research findings may be disseminated in academic papers or reports. If data need to be shared, only de-identified and minimal datasets will be provided; no personally identifiable information will ever be included.

5. Participation Requirements

Please read each item carefully and answer truthfully.

This survey is intended for respondents who are 18 years or older and regular metro users. If you do not meet these conditions or prefer not to participate, you may exit at any time without any obligation.

6. Informed Consent and Voluntary Participation

By proceeding to and submitting this questionnaire, you acknowledge that you have read and agreed to the above information and voluntarily choose to participate. You may exit the survey at any time. If, after submission, you wish to request the withdrawal of your data, please contact the research team at the email above; we will accommodate such requests where feasible.
