# Supplementary material for: Identifying and modeling built environment factors influencing cultural perception in metro stations: Evidence from central Shanghai
Source: PLoS One. 2025 Nov 6;20(11):e0334642. doi: 10.1371/journal.pone.0334642 (PMC12591495; doi:10.1371/journal.pone.0334642)
Supplement: S3 Appendix — (DOCX) [file pone.0334642.s003.docx]

**Survey on Cultural Perception and the Built Environment of Metro Stations in Central Shanghai**

**Introduction**

Hello, and thank you for participating in this survey. The questionnaire aims to understand your cultural perception of metro stations and your subjective evaluations of the station proper design and facilities, as well as station-area public cultural facilities. The study focuses on metro stations in central Shanghai, with a reference station-area radius of 500 m. Responses are anonymous and used only for academic research. Results will be reported in aggregate and will not include any personally identifiable information. The survey takes about 6–10 minutes, and you may stop at any time.

**Instructions**

Please answer with reference to the metro station you use most frequently.

All items are single-choice questions.

For items about nearby public cultural facilities, answer based on your subjective perception, not objective counts or map measurements.

If uncertain, answer according to your first instinct and try not to leave items blank.

Thank you again for your support. Submitting the questionnaire indicates that you have been informed about and agree to participate in this study.

**Q1 Please select your gender.**

□ Male

□ Female

**Q2 Please select your age group.**

□ Under 20

□ 20–30

□ 31–40

□ 41–50

□ Above 50

**Q3 Please select your highest level of education.**

□ High school or below

□ Associate or Bachelor’s degree

□ Master’s degree or above

**Q4 Which of the following metro stations do you use most frequently? [Single choice]**

□ JiaShan Lu

□ NanJingDong Lu

□ YuYuan

□ BeiXinJing

□ CaoBao Lu

□ YanChang Lu

□ ZiRanBoWuGuan

□ LaoXiMen

□ LuJiaBang Lu

□ LongHua

□ ShiLong Lu

□ SiPing Lu

**D1. I am satisfied with the size of this station’s entrances and exits.**

□ Very dissatisfied □ Dissatisfied □ Neutral □ Satisfied □ Very satisfied

**D2. I am satisfied with the decorative elements at this station’s entrances and exits.**

□ Very dissatisfied □ Dissatisfied □ Neutral □ Satisfied □ Very satisfied

**D3. I am satisfied with the signage design at this station’s entrances and exits.**

□ Very dissatisfied □ Dissatisfied □ Neutral □ Satisfied □ Very satisfied

**D4. I am satisfied with the public art at this station’s entrances and exits.**

□ Very dissatisfied □ Dissatisfied □ Neutral □ Satisfied □ Very satisfied

**D5. I am satisfied with the landscape design around this station’s entrances and exits.**

□ Very dissatisfied □ Dissatisfied □ Neutral □ Satisfied □ Very satisfied

**D6. I feel there are enough public cultural facilities within a 500 m radius of this station.**

□ Strongly disagree □ Disagree □ Neutral □ Agree □ Strongly agree

**D7.** **I think the types of public cultural facilities within a 500 m radius of this station are diverse.**

□ Strongly disagree □ Disagree □ Neutral □ Agree □ Strongly agree

**D8.** **I think the public cultural facilities within a 500 m radius of this station are well known in this area.**

□ Strongly disagree □ Disagree □ Neutral □ Agree □ Strongly agree

**D9. It is easy for me to reach public cultural facilities within a 500 m radius of this station.**

□ Strongly disagree □ Disagree □ Neutral □ Agree □ Strongly agree

**D10 I am satisfied with the public art inside the station.**

□ Very dissatisfied □ Dissatisfied □ Neutral □ Satisfied □ Very satisfied

**D11 I am satisfied with the cultural activities inside the station.**

□ Very dissatisfied □ Dissatisfied □ Neutral □ Satisfied □ Very satisfied

**D12 I am satisfied with the design of the station’s walls and columns (for example: color, materials, layout, etc.).**

□ Very dissatisfied □ Dissatisfied □ Neutral □ Satisfied □ Very satisfied

**D13 I am satisfied with the design of the station’s ceilings (for example: color, materials, layout, etc.).**

□ Very dissatisfied □ Dissatisfied □ Neutral □ Satisfied □ Very satisfied

**D14 I am satisfied with the design of the station’s floors (for example: color, materials, patterns, layout, etc.).**

□ Very dissatisfied □ Dissatisfied □ Neutral □ Satisfied □ Very satisfied

**D15 I am satisfied with the guide system inside the station.**

□ Very dissatisfied □ Dissatisfied □ Neutral □ Satisfied □ Very satisfied

**D16 I am satisfied with the supporting facilities inside the station, (for example: seating, lighting, etc.).**

□ Very dissatisfied □ Dissatisfied □ Neutral □ Satisfied □ Very satisfied

**D17 I am satisfied with the advertising facilities inside the station.**

□ Very dissatisfied □ Dissatisfied □ Neutral □ Satisfied □ Very satisfied

**D18 I think this metro station has a strong cultural atmosphere.**

□ Strongly disagree □ Disagree □ Neutral □ Agree □ Strongly agree

**D19** **I think this metro station has a strong sense of history.**

□ Strongly disagree □ Disagree □ Neutral □ Agree □ Strongly agree

**D20 I think this metro station has a strong design style and artistic atmosphere.**

□ Strongly disagree □ Disagree □ Neutral □ Agree □ Strongly agree

**D21 I think this metro station has a strong regional or local character.**

□ Strongly disagree □ Disagree □ Neutral □ Agree □ Strongly agree
